# Characteristics of Gender-Diverse Patients With Breast Cancer

**DOI:** 10.1001/jamanetworkopen.2026.24824

**Published:** 2026-07-23

**Authors:** Chandler S. Cortina, Ruta Brazauskas, Meghan R. Flanagan, Ellen D. Dill, Kathie-Ann P. Joseph, Alexandr Trifonov, Rita A. Mukhtar, Esther A. Kim, Olga Kantor, Gabi Barmettler, Kristen M. Rezak, Laura H. Rosenberger, Sumanas W. Jordan, Megan M. Perez, Rachel B. Jimenez, Alicia C. Smart, Katherine A. Kopkash, Tasha M. Hughes, Jasmine C. Walker, Meeghan A. Lautner, Emily L. Siegel, Danielle E. Brabender, Zahraa Al-Hilli, Michael R. Cassidy, Anna Weiss, Rachel L. McCaffrey, Marie C. Lee, Christodoulos Kaoutzanis, Khoa N. Nguyen, Jennifer D. Son, Austin D. Williams, Andrea Madrigrano, Sarah A. McLaughlin, Andrew E. Petroll, Melinda Stolley

**Affiliations:** 1 Division of Surgical Oncology, Department of Surgery, Medical College of Wisconsin, Milwaukee; 2 Medical College of Wisconsin Cancer Center, Milwaukee; 3 Division of Biostatistics, Medical College of Wisconsin, Milwaukee; 4 Department of Surgery, University of Washington, Seattle; 5 Division of Public Health Sciences, Fred Hutchinson Cancer Center, Seattle, Washington; 6 University of Washington School of Medicine, Seattle; 7 Departments of Surgery and Population Health, New York University (NYU) Grossman School of Medicine/NYU Langone Health, New York; 8 LGBTQ+ Care and Research Program, NYU Langone Perlmutter Cancer Center, NYU Grossman School of Medicine, New York; 9 Division of Surgical Oncology, Department of Surgery, University of California San Francisco, San Francisco; 10 Division of Plastic Surgery, Department of Surgery, University of California San Francisco, San Francisco; 11 Department of Surgery, Brigham and Women’s Hospital, Boston, Massachusetts; 12 Harvard Medical School, Boston, Massachusetts; 13 Division of Plastic, Maxillofacial, and Oral Surgery, Duke University Medical Center, Durham, North Carolina; 14 Department of Surgery, Duke University, Durham, North Carolina; 15 Division of Plastic and Reconstructive Surgery, Department of Surgery, Northwestern University, Chicago, Illinois; 16 Department of Radiation Oncology, Massachusetts General Hospital, Boston; 17 Department of Radiation Oncology, Brigham and Women’s Hospital, Boston, Massachusetts; 18 Department of Surgery, Northshore University HealthSystem, Evanston, Illinois; 19 University of Chicago Pritzker School of Medicine, Chicago, Illinois; 20 Department of Surgery, University of Michigan, Ann Arbor; 21 Division of Surgical Oncology, Department of Surgery, University of Wisconsin School of Medicine and Public Health, Madison; 22 Department of Surgery, University of Southern California, Los Angeles; 23 Breast Center, Integrated Surgical Institute, Cleveland Clinic, Cleveland, Ohio; 24 Section of Surgical Oncology Department of Surgery, Boston University Chobanian and Avedisian School of Medicine Boston Medical Center, Boston, Massachusetts; 25 Department of Surgery, University of Rochester, Rochester, New York; 26 Division of Surgical Oncology, Department of Surgery, Vanderbilt University Medical Center, Nashville, Tennessee; 27 Comprehensive Breast Program, Moffitt Cancer Center, Tampa, Florida; 28 Division of Plastic and Reconstructive Surgery, Department of Surgery, University of Colorado, Aurora; 29 University of Colorado School of Medicine, University of Colorado–Denver, Anschutz Medical Campus, Denver; 30 Department of Surgery, MedStar Montgomery and MedStar Georgetown University Hospital, Washington, DC; 31 Department of Surgical Oncology, Fox Chase Cancer Center, Philadelphia, Pennsylvania; 32 Department of Surgery, Rush University Medical Center, Chicago, Illinois; 33 Department of Surgery, Mayo Clinic, Jacksonville, Florida; 34 Division of Infectious Disease, Department of Medicine, Medical College of Wisconsin, Milwaukee; 35 Froedtert and the Medical College of Wisconsin Inclusion Health Clinic, Milwaukee; 36 Division of Hematology and Oncology, Department of Medicine, Medical College of Wisconsin, Milwaukee

## Abstract

**Importance:**

Limited granular data exist on breast cancer in transgender, nonbinary, and/or gender-diverse (TGD) individuals.

**Objective:**

To describe patient demographic and tumor clinicopathological variables, clinically relevant risk factors, method of breast cancer detection, treatment characteristics, and locoregional outcomes and to estimate a 5-year breast cancer–specific survival (BCSS).

**Design, Setting, and Participants:**

This cohort study obtained data on patients treated from 1990 to 2023 from 22 US academic medical centers using institutional databases, electronic medical records, and tumor registries. Included participants were TGD individuals 18 years or older with stage 0 to IV breast cancer. A comparative breast cancer cohort was selected from the 2016 to 2021 Surveillance, Epidemiology, and End Results Program (SEER) breast cancer dataset. Data were analyzed from January to October 2025.

**Exposure:**

Breast cancer.

**Main Outcomes and Measures:**

Estimated 5-year BCSS and locoregional recurrence rates. Wilcoxon rank sum and χ^2^ tests were used to compare continuous and categorical variables. Kaplan-Meier analysis was used to estimate 5-year BCSS.

**Results:**

A total of 112 TGD persons (median [IQR] age at diagnosis, 42.5 [36.5-51.0] years; 104 individuals [92.9%] were assigned female at birth) with breast cancer and 1 with bilateral disease were included in the analysis. Forty-three patients (38.4%) used gender-affirming hormone therapy before diagnosis. Among the entire cohort, 58 patients (51.8%) detected their own breast cancer, 68 (60.7%) had a familial breast cancer history, and 16 (14.3%) had a pathogenic germline variant. Tumors were predominantly hormone receptor positive (96 [85.7%]) and early stage (29 [25.7%] ductal carcinoma in situ [DCIS]; 51 [45.1%] stage I). The TGD cohort, compared with patients with breast cancer in the SEER dataset (N = 480 915), was younger (median [IQR] age, 42.5 [36.5-51.0] years vs 62.0 [52.0-72.0] years), had a higher proportion of persons assigned male at birth (8 [7.1%] vs 3522 [0.7%]), more often had progesterone receptor–positive disease (89 [79.5%] vs 335 491 [69.8%]), and presented with earlier-stage disease (eg, DCIS: 29 [25.9%] vs 79 604 [16.6%]). With a median (IQR) follow-up time for the TGD cohort of 38 (24-74) months, 5-year BCSS was favorable at 96.2% (95% CI, 85.3%-99.0%).

**Conclusions and Relevance:**

This cohort study of TGD patients with breast cancer found that this population was young and often detected their own tumors via self-examination, suggesting opportunities to improve early detection through screening for individuals at elevated risk. Although short-term BCSS appeared favorable, prospective research is essential to define evidence-based guidelines and elucidate long-term outcomes.

## Key Points

**Question:**

What are the methods of diagnosis and breast cancer–specific survival (BCSS) among transgender, nonbinary, and/or gender-diverse (TGD) individuals with breast cancer?

**Findings:**

In this cohort study of 112 TGD individuals with breast cancer, patients were generally young at diagnosis, and 51.8% of tumors were self-detected. With a median follow-up of 38 months, the estimated 5-year BCSS was 96.2%.

**Meaning:**

Although short-term BCSS was favorable, the cohort’s young age at diagnosis underscores the value of identifying individuals at elevated risk who may benefit from risk-reducing strategies and earlier breast cancer screening.

## Teaser

This cohort study identifies the demographic and tumor characteristics, disease detection methods, and survival outcomes for transgender, nonbinary, and/or gender-diverse patients with stage 0 to IV breast cancer.

## Introduction

A growing number of individuals identify as transgender, nonbinary, and/or gender diverse (TGD).^1^ Many TGD individuals use gender-affirming therapies such as gender-affirming hormone therapy (GAHT) with estrogen, progesterone, and/or testosterone, while some undergo gender-affirming surgery.^2^ Gender-affirming therapies provide several benefits, but there is a paucity of data on how gender-affirming therapies affect hormonally mediated cancers, such as breast cancer, in TGD persons.^3,4,5,6^

Several retrospective cohort studies of TGD persons with breast cancer have been conducted over the past decade, but they had limited sample sizes and outcomes data.^7,8,9,10^ One recent study of data from the National Cancer Database reported on a cohort of 252 TGD patients with breast cancer and found that these individuals were less likely to receive endocrine therapy for hormone receptor (HR)–positive disease and had inferior overall survival compared with matched cisgender patients.^11^ However, data on the method of diagnosis, family history, germline genetic testing, receipt of gender-affirming therapies, and decision-making are not available in the National Cancer Database, thereby limiting the study’s findings. Additional data are needed to elucidate the etiological factors underlying breast cancer outcome disparities to inform breast cancer screening and treatment guidelines for the TGD community.

To directly address these knowledge gaps, we created—to our knowledge—the first national multicenter cohort of TGD patients with breast cancer. We aimed to describe patient demographic and tumor clinicopathological variables, clinically relevant risk factors, method of breast cancer detection, treatment characteristics, and locoregional outcomes and to estimate a 5-year breast cancer–specific survival (BCSS). We hypothesized that TGD patients with breast cancer would be younger given their age distribution within the general population^1^ and thus would have a higher frequency of self-detected rather than screening-detected disease.

## Methods

We conducted a retrospective cohort study of TGD individuals diagnosed with stage 0 to IV breast cancer who were 18 years or older at the time of diagnosis and had available treatment and follow-up data from January 1, 1990, to December 31, 2023, across 22 centers in the US. The Medical College of Wisconsin (MCW) Institutional Review Board and the Institutional Review Boards of each contributing center approved this study and waived the informed consent requirement because deidentified data were used. A data use agreement was executed between MCW and each center. We followed the Strengthening the Reporting of Observational Studies in Epidemiology (STROBE) reporting guideline.^12^

Eligible patients at each center were first identified by searching institutional databases and tumor registries, depending on each center’s available resources. Next, each potentially eligible patient’s electronic medical record was thoroughly reviewed by a study team member to ensure cohort eligibility. TGD identity was defined as an individual whose sex assigned at birth was different from the sex and/or gender reported in the medical record (eg, a person assigned male at birth who identifies as a nonbinary transgender woman, a female, and/or a woman) or an individual who specified a gender different from their sex assigned at birth in the medical record (eg, a person assigned female at birth who identifies as a nonbinary transgender man, a man, or a male) on investigator review. Race and ethnicity were self-reported and collected from the electronic medical record. The racial and ethnic categories included Asian, Hispanic (all races), non-Hispanic Black, non-Hispanic White, multiracial, and other (American Indian and Alaska Native, Native Hawaiian, and Pacific Islander) or unknown. These data were included in this analysis to characterize the cohort and because these social constructs may reflect differential exposure to structural and health care factors associated with treatment patterns and clinical outcomes.

Investigators at each site were responsible for data collection, accuracy, and uploading anonymized data into a central registry (REDCap) where it was independently reviewed for accuracy. Data were extracted for 114 variables on patient demographic characteristics, gender-affirming therapy use, familial cancer history, tumor clinicopathological data, breast cancer treatment, survival, and follow-up. A patient’s last known status at time of data entry was recorded to examine disease recurrence and death. Data lock occurred in September 2024, and all data were analyzed at MCW from January to October 2025.

A comparative breast cancer cohort was selected from the 2016 to 2021 Surveillance, Epidemiology, and End Results Program (SEER) breast cancer dataset to explore how the TGD cohort compared with the larger general population with breast cancer. Patients in SEER were included if they were diagnosed with stage 0 to IV breast cancer and were excluded if diagnosed at time of autopsy.

### Statistical Analysis

Descriptive statistics are reported as numbers with percentages or medians with IQRs. Wilcoxon rank sum and χ^2^ tests were used to compare continuous and categorical variables, respectively. The Kaplan-Meier method was used to estimate 5-year BCSS, with breast cancer–related deaths treated as events and non–breast cancer–related deaths censored at time of occurrence. A 2-sided *P* ≤ .05 was considered statistically significant. Statistical analyses were conducted using SAS, version 9.4 (SAS Institute Inc).

## Results

The cohort included 112 TGD individuals, 1 of whom had bilateral disease resulting in a total of 113 breast cancers. The median (IQR) age at diagnosis was 42.5 (36.5-51.0) years. The cohort included 104 individuals (92.9%) assigned female at birth and 8 (7.1%) assigned male at birth, among whom the most common gender identities were nonbinary (50 [44.6%]), transgender man or man (28 [25.0%]), and genderqueer (20 [17.9%]) (Table 1). Seven patients (6.3%) self-identified as Asian, 4 (3.6%) as Hispanic, 13 (11.6%) as non-Hispanic Black, 82 (73.2%) as non-Hispanic White, and 3 (2.7%) as multiracial persons, with 3 (2.7%) identifying as other or unknown race and ethnicity. Sixty-seven patients (59.8%) had private insurance. Forty-eight individuals (42.9%) had a history of tobacco use, 18 (16.1%) had a history of alcohol use disorder, and 7 (6.3%) had a history of illicit drug use. Of those assigned female at birth, 64 (61.5%) were premenopausal and 33 (31.7%) had a prior pregnancy with 25 (24.0%) having given birth.

**Table 1.** Demographic Characteristics of TGD Patients With Breast Cancer at 22 US Centers, 1990-2023

Variable	TGD patients, No. (%)		
	Overall (N = 112)	Assigned female at birth (n = 104)	Assigned male at birth (n = 8)
Age, median IQR, y	42.5 (36.5-51.0)	42.0 (36.0-50.0)	55.0 (42.5-67.0)
BMI, median (IQR)	27 (23.0-31.5)	27 (23-31.5)	28.5 (23-31.5)
Sex assigned at birth			
Male	8 (7.1)	0	8 (100)
Female	104 (92.9)	104 (100)	0
Gender identity			
Transgender man	28 (25.0)	28 (26.9)	0
Transgender woman	5 (4.5)	0	5 (62.5)
Nonbinary	50 (44.6)	48 (46.2)	2 (25.0)
Genderqueer	20 (17.9)	20 (19.2)	0
Other	9 (8.0)	8 (7.7)	1 (12.5)
Sexual identity			
Straight	12 (10.7)	11 (10.6)	1 (12.5)
Gay	3 (2.7)	3 (2.9)	0
Lesbian	16 (14.3)	16 (15.4)	0
Bisexual	24 (21.4)	24 (23.1)	0
Pansexual	3 (2.7)	2 (1.9)	1 (12.5)
Asexual	1 (0.9)	1 (1.0)	0
Unknown	53 (47.3)	47 (45.2)	6 (75.0)
Race and ethnicity^a^			
Asian	7 (6.3)	7 (6.7)	0
Hispanic, all races	4 (3.6)	4 (3.8)	0
Non-Hispanic Black	13 (11.6)	10 (9.6)	3 (37.5)
Non-Hispanic White	82 (73.2)	78 (75.0)	4 (50.0)
Multiracial	3 (2.7)	2 (1.9)	1 (12.5)
Other or unknown^b^	3 (2.7)	3 (2.9)	0
Insurance status			
Private	67 (59.8)	64 (61.5)	3 (37.5)
Medicare	24 (21.4)	21 (20.2)	3 (37.5)
Medicaid	16 (14.3)	15 (14.4)	1 (12.5)
Other or unknown^c^	5 (4.5)	4 (3.9)	1 (12.5)
Menopause status (n = 104)			
Premenopause	64 (61.5)	64 (61.5)	NA
Postmenopause	26 (25.0)	26 (25.0)	NA
Perimenopause	5 (4.8)	5 (4.8)	NA
Unknown	9 (8.7)	9 (8.7)	NA
History of OCP use (n = 104)			
Yes	39 (37.5)	39 (37.5)	NA
No	63 (60.6)	63 (60.6)	NA
Unknown	2 (1.9)	2 (1.9)	NA
History of GAHT prior to diagnosis			
Yes	43 (38.4)	36 (34.6)	7 (87.5)
Estrogen	8	1	7
Progesterone	2	1	1
Testosterone	35	35	0
Other^d^	2	0	2
No	69 (61.6)	68 (65.4)	1 (12.5)
History of gender-affirming top surgery prior to diagnosis			
Yes, GACMS	12 (10.7)	12 (11.5)	0
Yes, breast augmentation	0	0	0
No	100 (89.3)	92 (88.5)	8 (100)
Method of BC diagnosis			
Self-examination or symptoms	58 (51.8)	52 (50.0)	6 (75.0)
Clinician detected on physical examination	4 (3.6)	4 (3.8)	0
Screening mammography	31 (27.7)	29 (27.9)	2 (25.0)
Incidental finding on other imaging	3 (2.7)	3 (2.9)	0
Incidental finding on pathology for GACMS	15 (13.4)	15 (14.4)	0
Unknown	1 (0.9)	1 (1.0)	0

Abbreviations: BC, breast cancer; BMI, body mass index (calculated as weight in kilograms divided by height in meters squared); GACMS, gender-affirming chest masculinization surgery; GAHT, gender-affirming hormone therapy; NA, not applicable; OCP, oral contraceptive pill; TGD, transgender, nonbinary, and/or gender-diverse.

^a^ Race and ethnicity were self-reported.

^b^ Other race includes American Indian and Alaska Native, Native Hawaiian, and Pacific Islander.

^c^ Other or unknown insurance status includes self-pay and missing data in the electronic medical record.

^d^ Other hormones include gonadotropin-releasing hormone agonists, antiandrogens (eg, spironolactone), and other GAHT agents that were not estrogen, progesterone, or testosterone.

### Familial History and Genetics

Overall, 68 of 112 individuals (60.7%) had a familial breast cancer history. Thirty-nine TGD individuals (34.8%) had a first-degree relative with breast cancer and 51 (45.5%) had a second-degree relative with breast cancer. One hundred patients (89.3%) were offered genetic counseling, of whom 85 (85.0%) met with a genetic counselor and 84 (84.0%) underwent pathogenic germline testing. Of the 84 patients who underwent testing, 16 (19.0%) had a pathogenic germline variant, 12 (14.3%) had a variant of uncertain significance, and 56 (66.7%) had no pathogenic variants detected. Among the entire cohort (n = 112), 16 patients (14.3%) had an identifiable pathogenic germline variant, of which the most common were *BRCA1* (3 [18.8%]) and *BRCA2* (4 [25.0%]) (Table 2). Two patients met with a genetic counselor prior to their breast cancer diagnosis due to a known familial pathogenic germline variant. Both patients were found to harbor pathogenic germline variants in *BRCA2* and *TP53,* respectively, and the patient with known *BRCA2* variant was enrolled in a high-risk screening program before diagnosis.

**Table 2.** TGD Patients With Breast Cancer With Either a Pathogenic Variant or Variant of Uncertain Significance

Pathogenic germline variant	TGD patients, No. (%)
**Germline variant (n = 16)**	
*BARD1*	1 (6.3)
*BRCA1*	3 (18.8)
*BRCA2*	4 (25.0)
*BRIP1*	1 (6.3)
*CHEK2*	1 (6.3)
*MITF*	1 (6.3)
*NF1*	1 (6.3)
*PALB2*	1 (6.3)
*POLE*	1 (6.3)
*STK11*	1 (6.3)
*TP53*	1 (6.3)
**Germline VUS (n = 17)^a^**	
*ALK*	1 (5.9)
*APC*	1 (5.9)
*ATM*	2 (11.8)
*BRCA2*	1 (5.9)
*CHEK2*	1 (5.9)
*DICER1*	1 (5.9)
*BARD1*	1 (5.9)
*FANCC*	1 (5.9)
*FLCN*	1 (5.9)
*GALNT12*	1 (5.9)
*MAX*	1 (5.9)
*POLE*	1 (5.9)
*RB1*	1 (5.9)
*RAD51C*	1 (5.9)
*RECQL4*	1 (5.9)
*SMARCA4*	1 (5.9)

Abbreviations: TGD, transgender, nonbinary, and/or gender-diverse; VUS, variant of uncertain significance.

^a^ Five patients had 2 or more VUS identified.

Among the 8 TGD persons assigned male at birth, 3 (37.5%) had a first-degree relative (mother) with a history of breast cancer and 7 (87.5%) were referred to genetic counseling. Of these 7 patients, all underwent testing, with 1 found to have a pathogenic *BRCA2* germline variant and 1 with a pathogenic *PALB2* germline variant, resulting in a 25% prevalence rate of pathogenic germline variant for persons assigned male at birth.

### Gender-Affirming Therapy

A total of 43 patients (38.4%) used GAHT prior to their diagnosis (median [IQR] GAHT duration, 3 [1-11] years). Eight of 112 patients (7.1%) received GAHT with estrogen, 2 (1.8%) with progesterone, and 35 (31.3%) with testosterone. Of the 8 TGD persons assigned male at birth, 7 (87.5%) used gender-affirming estrogen before diagnosis and 1 (12.5%) used gender-affirming progesterone prior to diagnosis. Two patients continued GAHT after diagnosis. Of the 104 TGD persons assigned female at birth, 12 (11.5%) underwent gender-affirming chest masculinization surgery (GACMS) prior to their diagnosis. Additionally, these patients underwent GACMS more often compared with those who had not used GAHT (9 of 35 [25.7%] vs 3 of 69 [4.3%]; *P* = .003). Five individuals (4.5%) underwent gender-affirming bottom surgery prior to diagnosis: 3 were assigned female at birth (2 of whom had oophorectomy), and 2 were assigned male at birth (both had orchiectomy).

### Breast Cancer Screening and Method of Detection

While 58 patients (51.8%) detected their own tumors by self-examination or symptoms, 31 (27.7%) found out about their disease from screening mammography, 15 (13.4%) incidentally from post-GACMS pathology, 4 (3.6%) from a physical examination by a clinician, and 3 (2.7%) incidentally from nonbreast imaging (Table 1). Fifty-eight patients (51.8%) had a screening mammogram within 10 years prior to their breast cancer diagnosis. Of the 47 individuals with a first-degree familial breast cancer history and/or a pathogenic germline variant, 22 (46.8%) found out about their tumors through self-detection, 14 (29.8%) through screening mammography, 6 (12.8%) incidentally through post-GACMS pathology, 3 (6.4%) incidentally through nonbreast imaging, and 2 (4.3%) through a clinician.

Among the 104 individuals assigned female at birth, 56 (53.9%) had undergone screening mammography in their lifetime. Of the 12 patients who had GACMS before diagnosis, 5 (41.7%) had a screening mammogram before GACMS compared with 51 (55.4%) of the 92 persons who did not have GACMS (*P* = .54). Five persons assigned female at birth underwent screening mammography after GACMS, and there was 1 cancer detected. In 51 persons who did not have GACMS and underwent screening mammography, 25 (49.0%) had breast cancer that was detected via screening. Screening mammography was performed in 2 (25.0%) of the 8 individuals assigned male at birth, and their cancer was detected on screening; both patients were in their eighth decade of life and receiving GAHT.

### Breast Cancer Data

One hundred thirteen tumors were either ductal carcinoma in situ (DCIS; 33 [29.2%]) or cT1 to T2 (72 [63.5%]), and 83 tumors (73.5%) were clinically node-negative (cN0) (Table 3). Tumors were predominantly HR positive (96 [85.7%]) and early stage (29 [25.7%] DCIS; 51 [45.1%] stage I); however, 10 (8.8%) were *ERBB2* (formerly *HER2*) positive or amplified and 4 (3.5%) were triple-negative breast cancer. Of the 112 TGD patients, 88 (78.6%) underwent upfront surgery, 22 (19.6%) received neoadjuvant systemic therapy (20 chemotherapy and 2 endocrine therapy), and 1 patient (0.9%) received systemic therapy only due to de novo stage IV disease.

**Table 3.** Tumor Clinicopathological Variables and Treatment Characteristics of TGD Patients With Breast Cancer

Variable	TGD patients, No. (%)		
	Overall	Assigned female at birth	Assigned male at birth
**Tumor clinicopathological variables**			
No. of tumors	113	105	8
Clinical T category			
Tis	33 (29.2)	32 (30.5)	1 (12.5)
T1	32 (28.1)	32 (30.5)	0
T2	40 (35.4)	35 (33.3)	5 (62.5)
T3	6 (5.3)	4 (3.8)	2 (25.0)
T4	2 (1.8)	2 (1.9)	0
Clinical N category			
N0	83 (73.5)	79 (75.2)	4 (50.0)
N1	24 (21.2)	21 (20.0)	3 (37.5)
N2	4 (3.5)	3 (2.9)	1 (12.5)
N3	1 (0.9)	1 (1.0)	0
Unknown	1 (0.9)	1 (1.0)	0
Clinical M category			
M0	109 (96.5)	101 (96.2)	8 (100)
M1	4 (3.5)	4 (3.8)	0
ER status			
Positive	96 (85.0)	88 (83.8)	8 (100)
Negative	11 (9.7)	11 (10.5)	0
Unknown	6 (5.3)	6 (5.7)	0
PR status			
Positive	90 (79.6)	83 (79.0)	7 (87.5)
Negative	14 (12.4)	13 (12.4)	1 (12.5)
Unknown	9 (8.0)	9 (8.6)	0
*ERBB2* status			
Positive or amplified	10 (8.8)	7 (6.7)	3 (37.5)
Negative or nonamplified	81 (71.7)	76 (72.4)	5 (62.5)
Unknown^a^	22 (19.5)	22 (21.0)	0
Molecular subtype			
DCIS	29 (25.7)	28 (26.7)	1 (12.5)
Luminal A/B	70 (61.9)	65 (61.9)	5 (62.5)
*ERBB2* enriched	9 (8.0)	7 (6.7)	2 (25.0)
TNBC	4 (3.5)	4 (3.8)	0
Unknown	1 (0.9)	1 (1.0)	0
AJCC 8th Disease stage			
0	29 (25.7)	28 (26.7)	1 (12.5)
I	51 (45.1)	48 (45.7)	3 (37.5)
II	23 (20.4)	22 (21.0)	1 (12.5)
III	6 (5.3)	5 (4.8)	1 (12.5)
IV	4 (3.5)	2 (1.9)	2 (25.0)
Surgical breast or chest treatment			
Lumpectomy	31 (27.7)	27 (25.7)	4 (50.0)
Mastectomy	69 (61.6)	66 (62.9)	4 (50.0)
Completion mastectomy or wide local excision^b^	8 (7.2)	8 (7.6)	NA
BC fully removed during GACMS	3 (2.7)	3 (2.9)	NA
Did not undergo surgery	1 (0.9)	1 (1.0)	0
Surgical axillary staging			
Omission	28 (24.8)	25 (23.8)	3 (37.5)
SLNB	66 (58.4)	63 (60.0)	3 (37.5)
SLNB followed by ALND	9 (8.0)	8 (7.6)	1 (12.5)
ALND	9 (8.0)	8 (7.6)	1 (12.5)
Did not receive surgery	1 (0.9)	1 (1.0)	0
patients			
**Treatment characteristics**			
No. of patients	112	104	8
Received adjuvant radiotherapy			
Yes	46 (41.1)	42 (40.4)	4 (50.0)
No	66 (58.9)	62 (59.6)	4 (50.0)
Received chemotherapy			
Yes	46 (41.1)	41 (39.4)	5 (62.5)
No	66 (58.9)	63 (60.6)	3 (37.5)
Recommended to receive endocrine therapy			
Yes	79 (70.5)	73 (70.2)	6 (75.0)
No	33 (29.5)	31 (29.8)	2 (25.0)
Received endocrine therapy			
Yes	64 (57.1)	60 (57.7)	4 (50.0)
No	48 (42.9)	44 (42.3)	4 (50.0)

Abbreviations: AJCC, American Joint Committee on Cancer *Cancer Staging Manual*, 8th ed; ALND, axillary lymph node dissection; BC, breast cancer; DCIS, ductal carcinoma in situ; ER, estrogen receptor; GACMS, gender-affirming chest masculinization surgery; NA, not applicable; PR, progesterone receptor; SLNB, sentinel lymph node biopsy; TGD, transgender, nonbinary, and/or gender-diverse; TNBC, triple-negative breast cancer.

^a^ Unknown *ERBB2* status is based on persons with DCIS and did not have *ERBB2* analysis.

^b^ For patients who already had GACMS.

Sixty-nine patients (61.6%) were treated with mastectomy, while 31 (27.7%) underwent lumpectomy, and 8 (7.2%) were treated with completion mastectomy or wide local excision after prior GACMS. Three tumors (2.7%) were removed entirely during GACMS, and 1 tumor was not removed due to stage IV disease. There was no difference in surgery type by sex assigned at birth for those undergoing initial lumpectomy or mastectomy (Table 4). Of the 69 TGD patients who underwent mastectomy, 44 (63.8%) did not have postmastectomy breast reconstruction (eTable 1 in Supplement 1). Postmastectomy radiotherapy was performed in 22 patients (31.9%), primarily due to nodal disease burden. Of the 31 patients who had lumpectomy, 22 (71.0%) received adjuvant radiotherapy and, of the 8 patients who had a completion mastectomy or wide local excision after GACMS, only 2 (25.0%) received radiotherapy. In the 3 patients who had their disease detected incidentally on pathology for GACMS, all disease was resected to negative margins at the time of GACMS.

**Table 4.** Breast or Chest Operation Type by Sex Assigned at Birth

Sex assigned at birth	TGD patients, No. (%)		*P* value
	Breast-conserving surgery	Mastectomy	
Male (n = 8)	4 (50.0)	4 (50.0)	.22
Female (n = 100)	27 (27.0)	73 (73.0)^a^	

Abbreviation: TGD, transgender, nonbinary, and/or gender-diverse.

^a^ Includes 5 patients who underwent completion mastectomy or wide local excision after previous gender-affirming chest masculinization surgery.

Of the 112 TGD patients, 46 (41.1%) received systemic chemotherapy. Ninety-six of 112 patients (85.7%) had HR-positive disease, and 76 (67.8%) were recommended to receive endocrine therapy. Of these 76 patients, 64 (84.2%) received endocrine therapy and 13 (17.1%) did not. Endocrine therapy receipt did not differ by sex assigned at birth (4 [66.7%] for those assigned male at birth vs 59 [84.3%] for those assigned female at birth; *P* = .27) (Table 5). Additionally, of the 96 patients with HR-positive disease, 37 (38.5%) received GAHT: 20 started GAHT before their diagnosis and stopped after diagnosis, 8 started GAHT before their diagnosis and continued after diagnosis, and 9 started GAHT after their diagnosis. In total, 17 of the 96 patients (17.7%) with HR-positive disease received GAHT after their diagnosis, and 9 of the 17 (52.9%) also received endocrine therapy.

**Table 5.** Endocrine Therapy Receipt by Sex Assigned at Birth for TGD Patients Who Were Recommended to Receive Endocrine Therapy and Patients With Only Hormone Receptor–Positive Disease

Sex assigned at birth	TGD patients, No./total No. (%)		*P* value
	Received endocrine therapy	Did not receive endocrine therapy	
**Endocrine therapy recommendation**			
Recommended			.32
Male	4/6 (66.7)	2/6 (33.3)	
Female^a^	60/73 (82.2)	13/73 (17.8)	
Only HR-positive disease			
Male	4/6 (66.7)	2/6 (33.3)	.27
Female	59/70 (84.3)	11/70 (15.7)	

Abbreviations: HR, hormone receptor; TGD, transgender, nonbinary, and/or gender-diverse.

^a^ Three patients were recommended to take endocrine therapy despite having HR-negative disease.

### SEER Data and TGD Outcomes

The TGD cohort compared with patients with breast cancer in SEER (n = 480 915) were younger (median [IQR] age, 42.5 [36.5-51.0] years vs 62.0 [52.0-72.0] years), more often self-identified as non-Hispanic White (82 [73.2%] vs 306 501 [63.7%]), and had more cases of progesterone receptor–positive disease (89 [79.5%] vs 335 491 [69.8%]) (eTable 2 in Supplement 1). Additionally, the TGD cohort had a higher proportion of persons assigned male at birth than the SEER cohort (8 [7.1%] vs 3522 [0.7%]). TGD patients also had more cases of early-stage disease (DCIS: 29 [25.9%] vs 79 604 [16.6%]; stage I: 50 [44.6%] vs 241 454 [50.2%]) and luminal disease (69 [61.6%] vs 284 201 [59.1%]) compared with patients in SEER (eTable 2 in Supplement 1).

With a median (IQR) follow-up of 38 (24-74) months, 12 TGD patients (10.7%) had a locoregional recurrence with a median (IQR) time to locoregional recurrence of 61 (39-101) months. Regarding 5-year locoregional recurrence, there was no statistical difference in locoregional recurrence rates in patients with HR-positive disease who stopped or continued GAHT after their diagnosis vs those who never used GAHT (3 of 37 [8.1%] vs 7 of 59 [11.9%]) or between patients with HR-positive disease who were recommended and received endocrine therapy and patients who did not (9 of 63 [14.3%] vs 1 of 13 [7.7%]). Of the 31 patients who underwent lumpectomy, 9 (29.0%) omitted adjuvant radiotherapy. There was no statistical difference in 5-year locoregional recurrence in patients who underwent lumpectomy and received radiotherapy vs those who received lumpectomy and omitted radiotherapy (3 of 22 [13.6%] vs 0 of 9 [0%]).

Of the 17 TGD individuals with HR-positive disease who used GAHT after their diagnosis, 3 (17.6%) experienced a locoregional recurrence. Of the 79 individuals with HR-positive disease who did not receive GAHT after their diagnosis, 7 (8.8%) had a locoregional recurrence. The locoregional recurrence rate by GAHT receipt for those with HR-positive disease was not statistically different.

Two patients developed a distant recurrence at time of last contact, and both subsequently died of metastatic breast cancer. Five-year BCSS for the TGD cohort was favorable at 96.2% (95% CI, 85.3%-99.0%).

## Discussion

This large multicenter cohort study of TGD patients with breast cancer identified that TGD individuals were younger and more frequently assigned male at birth compared with the general population with breast cancer in SEER. Additionally, we observed an elevated pathogenic germline variant rate and low breast cancer screening rates, factors likely associated with the self-detection of most disease. While there was no difference in prognostic stage between the TGD and SEER patients and short-term BCSS was favorable, 10.7% of patients had a locoregional recurrence and 29.0% omitted radiotherapy after breast-conserving surgery, indicating an opportunity to increase receipt of guideline-concordant care. Our findings also suggest that breast cancer risk factors may be different in the TGD population; thus, there may be a role for tailored screening, regardless of sex assigned at birth, based on baseline breast cancer risk factors. Prospective data are ultimately needed to inform evidence-based screening and treatment guidelines and to identify long-term oncologic and patient-reported outcomes in the context of gender-affirming care use.

Increasing data reveal lower rates of breast cancer screening among TGD individuals, despite an increase in entities providing screening recommendations for the TGD community.^13,14,15,16,17,18,19,20,21,22^ In a single-institution retrospective cohort, less than 50% of TGD patients eligible for screening mammography underwent screening compared with 77% of cisgender women during the same period.^23^ Survey work has identified that many TGD patients are unaware that TGD-specific breast cancer screening guidelines exist and less than half of those 40 years or older report ever having had a screening mammogram.^24^ Additionally, varying rates of breast cancer screening adherence are seen depending on gender identity and age.^25^ While guidelines and clinicians may recommend breast cancer screening for TGD patients, not all insurance policies will cover breast imaging for TGD persons given the limited screening data available for this population.^26^ While our cohort was young, many had a familial breast cancer history, highlighting the value of refining screening guidelines and policy coverage, especially for TGD individuals with an elevated breast cancer risk.

Assessing an individual’s risk for breast cancer, especially among those with a known family history, is increasingly relevant when TGD individuals are considering GACMS. While GACMS aims to remove most breast tissue to provide a masculine chest, it is not generally considered to be surgically equivocal to a mastectomy, which focuses on removal of all breast tissue for the purpose of breast cancer risk reduction and/or treatment.^27,28,29,30^ In persons with a family history, genetic counseling and a personalized breast cancer risk calculation should be performed prior to GACMS to ensure that those with a pathogenic germline variant or an elevated breast cancer risk can consider oncologic risk-reducing mastectomies, as opposed to conventional GACMS alone, to minimize future breast cancer risk.^27,28,29,30^ Regardless of risk, persons who meet breast cancer screening criteria should undergo screening prior to GACMS and even if preoperative imaging is within normal limits. All tissue removed during GACMS should be routinely sent for pathological evaluation given that 13.4% of patients in this study had their cancer detected incidentally through pathological finding at the time of GACMS. Prior studies have also found that up to 6.4% of GACMS pathology specimens have atypia, which can inform surveillance and risk-reduction options after GACMS.^31,32,33,34^

The complexities associated with GAHT and breast cancer risk and adjuvant treatment for patients with HR-positive disease are largely underexplored. Nationwide data from the Netherlands suggest that GAHT with testosterone appears to reduce breast cancer risk in transgender men, whereas GAHT with estrogen may increase breast cancer risk in transgender women, similar to the risk profiles observed in cisgender individuals based on their endogenous hormone production.^8^ However, data also suggest that the risk of breast cancer from GAHT with testosterone is likely more nuanced than observed in the Dutch cohorts.^35^ Given our cohort’s design, we were not able to ascertain how receipt of GAHT directly contributes to breast cancer risk in TGD persons, and larger prospective cohorts are needed to understand the potential risk of GAHT for the US population. Additionally, the careful balance in optimizing adjuvant therapy for TGD patients with HR-positive disease who use GAHT requires careful attention in future work.^23,36,37^ Cohort data from the National Cancer Database identified that TGD patients with HR-positive disease were less likely to receive endocrine therapy for HR-positive disease compared with cisgender patients.^11^ In this series, we observed that 17.1% of patients with HR-positive disease declined endocrine therapy, and some actually continued or began GAHT after their diagnosis. Future patient-centered trials will be required to examine both oncologic and patient-reported outcomes for those with HR-positive breast cancer and using adjuvant endocrine therapy and GAHT to define best practices and optimize adherence to guideline-concordant care.^38,39^ While no differences in locoregional recurrence based on endocrine therapy or radiotherapy receipt were observed in this cohort, 29% omitted radiotherapy after lumpectomy. Future qualitative work is required to elucidate the omission of these therapies.

Some of the study findings may be explained through the lens of gender identity. For example, many patients who underwent mastectomy did not undergo postmastectomy breast reconstruction, which is likely associated with the large number of patients in our cohort who were assigned female at birth and may have desired a flat or masculine chest appearance as part of their breast cancer surgery. While no statistical difference was seen in surgical choice by sex assigned at birth in the TGD cohort, this comparison was likely limited by the low number of patients assigned male at birth. Future work using both the validated BREAST-Q and GENDER-Q^40,41,42^ measurement tools to collect patient-reported surgical outcomes will be instrumental in assessing patient satisfaction and quality of life in TGD patients after undergoing surgical treatment for breast cancer. Notably, 14.3% of our TGD cohort had a pathogenic germline variant, higher than the 5.0% prevalence observed in modern cohorts of cisgender women with breast cancer.^43^ This elevated rate of pathogenic germline variant may be attributed to the younger age of our cohort and the higher rates of younger populations identifying as TGD,^1,44^ or it may be associated with higher testing rates at the centers that participated in this cohort study.

### Strengths and Limitations

To our knowledge, this study is the most granular large cohort of TGD patients with breast cancer. Additionally, it identifies novel opportunities for future research aimed at reducing breast cancer disparities in the TGD population and advancing the development of evidence-based guidelines.

Despite the significance of its findings, this study has methodological limitations. First, due to the short median follow-up of 38 months, a limited number of events have occurred, requiring a longer follow-up. Second, some TGD patients with breast cancer during the study period may have been unintentionally excluded due to historic limitations in documenting gender separate from sex assigned at birth within medical records and datasets, which may disproportionally affect those with historically marginalized intersectional identities such as older age or those belonging to a racial and/or ethnic minority group. Third, while we collected data on the use of GAHT with estrogen, testosterone, and progesterone, several hormone-modulating treatments could also be used that were not captured in our dataset.^44,45^ Fourth, screening guidelines varied during the study, which limited additional analyses regarding screening uptake in our cohort. Fifth, the SEER dataset cannot identify endocrine therapy uptake except with linkage to SEER-Medicare (predominantly patients aged ≥65 years); therefore, this uptake was not examined. Sixth, differences in age at diagnosis, tumor characteristics, and BCSS between the TGD and SEER cohorts should not be interpreted as population-level data. Finally, many participating centers are tertiary academic medical centers, which limits generalizability of the findings.

## Conclusions

In this cohort of TGD individuals with breast cancer in the US, patients were generally young and most tumors were self-detected. These findings highlight the value of identifying individuals at high risk for breast cancer who may benefit from risk-reducing measures and earlier breast cancer screening. While BCSS was favorable, further strategic research is necessary to ascertain breast cancer risk and its association with gender-affirming therapies, inform evidence-based TGD breast cancer screening and treatment guidelines, and elucidate long-term oncologic and patient-reported outcomes.

## Notes

Supplement 1. **eTable 1.** Mastectomy Type for TGD Patients in the Cohort Who Underwent Mastectomy (n = 69) **eTable 2.** Baseline Patient Demographics, Tumor Clinicopathologic, and Treatment Variables of the TGD Cohort and the SEER 2016–2021 Breast Cancer Cohort

Supplement 2.
Data Sharing Statement
