# Error in the Byline

**DOI:** 10.1001/jamanetworkopen.2026.35940

**Published:** 2026-08-27

In the Original Investigation titled “Characteristics of Gender-Diverse Patients With Breast Cancer”^1^ published on July 23, 2026, there was an error in the byline. Dr Joseph’s first name was misspelled. Her name should appear as Kathie-Ann P. Joseph, MD, MPH. This article was corrected.
