# Supplementary material for: Characteristics of Gender-Diverse Patients With Breast Cancer
Source: JAMA Netw Open. 2026 Jul 23;9(7):e2624824. doi: 10.1001/jamanetworkopen.2026.24824 (PMC13397088; doi:10.1001/jamanetworkopen.2026.24824)
Supplement: Supplement 1. — eTable 1. Mastectomy Type for TGD Patients in the Cohort Who Underwent Mastectomy (n = 69) eTable 2. Baseline Patient Demographics, Tumor Clinicopathologic, and Treatment Variables of the TGD Cohort and the SEER 2016–2021 Breast Cancer Cohort [file jamanetwopen-e2624824-s001.pdf]

Supplemental Online Content

Cortina CS, Brazauskas R, Flanagan MR, et al. Characteristics of gender-diverse patients with breast cancer. *JAMA Netw Open*. 2026;9(7):e2624824. doi:10.1001/jamanetworkopen.2026.24824

eTable 1. Mastectomy Type for TGD Patients in the Cohort Who Underwent Mastectomy (n=69)

eTable 2. Baseline Patient Demographics, Tumor Clinicopathologic, and Treatment Variables of the TGD Cohort and the SEER 2016–2021 Breast Cancer Cohort

This supplemental material has been provided by the authors to give readers additional information about their work.

© 2026 Cortina CS et al. *JAMA Network Open.*

eTable 1. Mastectomy type for TGD patients in the cohort who underwent mastectomy (n=69)

Mastectomy Type	n=69<br>n (%)
Unilateral Simple	8 (11.6%)
Bilateral Simple	22 (31.9%)
Bilateral Simple with Free Nipple Graft	14 (20.3%)
Unilateral Skin-Sparing with Reconstruction	3 (4.4%)
Bilateral Skin-Sparing with Reconstruction	16 (23.2%)
Unilateral Nipple-Sparing with Reconstruction	1 (1.5%)
Bilateral Nipple-Sparing with Reconstruction	5 (7.3%)

44/69 (63.8%) had no recon

© 2026 Cortina CS et al. *JAMA Network Open.*

eTable 2. Baseline patient demographics, tumor clinicopathologic, and treatment variables of the TGD cohort and the SEER 2016–2021 breast cancer cohort.

Variables	TGD Cohort N=112	SEER Cohort N=480,915
**Age (years), Median IQR**	42.5 (36.5–51.0)	62.0 (52.0–72.0)
	**n (%)**	**n (%)**
**Sex Assigned at Birth**		
Female	104 (92.9)	477,393 (99.3)
Male	8 (7.1)	3,522 (0.7)
**Race/Ethnicity**		
Asian	7 (6.3)	51,492 (10.7)
Hispanic	4 (3.6)	63,642 (13.2)
NH Black	13 (11.6)	52,337 (10.9)
NH White	82 (73.2)	306,501 (63.7)
Other	3 (2.7)	2,879 (0.6)
Unknown	3 (2.7)	4,064 (0.8)
**AJCC T Category**		
Tis	33 (29.5)	53,038 (11.0)
T1	31 (27.7)	153,711 (32.0)
T2	40 (35.7)	91,203 (19.0)
T3	6 (5.4)	19,906 (4.1)
T4	2 (1.8)	14,741 (3.1)
Unknown	0	148,316 (30.8)
**AJCC N Category**		
N0	82 (73.2)	271,500 (56.5)
N1	24 (21.4)	68,751 (14.3)
N2	4 (3.6)	9,832 (2.0)
N3	1 (0.9)	8,980 (1.9)
Unknown	1 (0.9)	121,852 (25.3)
**AJCC M Category**		
M0	108 (96.4)	450,875 (93.8)
M1	4 (3.6)	23,615 (4.9)
Unknown	0 (0.0)	6,425 (1.3)
**ER Status**		
Negative	11 (9.8)	70,844 (14.7)
Positive	95 (84.8)	393,331 (81.8)
Missing	6 (5.4)	16,740 (3.5)
**PR Status**		
Negative	14 (12.5)	117,206 (24.4)
Positive	89 (79.5)	335,491 (69.8)
Unknown	9 (8.0)	28,218 (5.9)
**HER2 Status**		
Negative/Nonamplified	80 (71.4)	327,918 (68.2)
Positive/Amplified	10 (8.9)	55,454 (11.5)
Unknown	22 (19.6)	97,543 (20.3)
**Molecular Subtype**		
DCIS	29 (25.9)	79,604 (16.6)
Luminal A/B	69 (61.6)	284,201 (59.1)
HER2 Enriched	9 (8.0)	54,258 (11.3)
TNBC	4 (3.6)	40,106 (8.3)
Unknown	1 (0.9)	22,746 (4.7)
**Prognostic Stage**		
0	29 (25.9)	77,851 (16.2)
I	50 (44.6)	241,454 (50.2)
II	23 (20.5)	74,812 (15.6)
III	6 (5.4)	29,432 (6.1)
IV	4 (3.6)	23,680 (4.9)

© 2026 Cortina CS et al. *JAMA Network Open.*

Variables	TGD Cohort N=112	SEER Cohort N=480,915
Unknown	0 (0.0)	33,686 (7.0)
**Breast/Chest Surgery**		
Lumpectomy	31 (27.7)	265,981 (55.3)
Mastectomy/GACMS^	77 (68.9)	163,023 (33.9)
N/A or Did Not Undergo Surgery	4 (3.6)	49,997 (10.4)
Unknown	0 (0)	1,914 (0.4)
**Post-Mastectomy Reconstruction**		
No	44 (63.8)	111,495 (68.4)
Yes	25 (36.2)	51,528 (31.6)
**Chemotherapy**		
No	66 (58.9)	332,908 (69.2)
Yes	46 (41.1)	148,007 (30.8)
**Radiation Therapy**		
No	66 (58.9)	241,883 (50.3)
Yes	46 (41.1)	239,032 (49.7)

Key:

NH=non-Hispanic
GACMS= gender-affirming chest masculinization surgery
*=Other race includes multiracial, Native Hawaiian, Pacific Islander, American Indian, or Native Alaskan
^= includes TGD patients who underwent completion mastectomy after GACMS or wide local excision after GACMS.
TNM categories based on AJCC 8th edition.

© 2026 Cortina CS et al. *JAMA Network Open.*
