# Supplementary material for: Characteristics of Gender-Diverse Patients With Breast Cancer
Source: JAMA Netw Open. 2026 Jul 23;9(7):e2624824. doi: 10.1001/jamanetworkopen.2026.24824 (PMC13397088; doi:10.1001/jamanetworkopen.2026.24824)
Supplement: Supplement 2. — Data Sharing Statement [file jamanetwopen-e2624824-s002.pdf]

Data Sharing Statement

Cortina. Characteristics of Gender-Diverse Patients With Breast Cancer. *JAMA Netw Open*. Published July 23, 2026. doi:10.1001/jamanetworkopen.2026.24824

Data
Data available: Yes
Data types: Deidentified participant data
How to access data: Data will be available upon reasonable request for research purposes after July 1, 2027
When available: beginning date: 07-01-2027

Supporting Documents
Document types: None

Additional Information
Who can access the data: Data will be available upon reasonable request for research purposes after July 1, 2027
Types of analyses: Approved reach questions that can be answered by the dataset.
Mechanisms of data availability: After proposal approval and signed DUA.
